# Giant thyroid metastasis originating from renal carcinoma: a case report

**DOI:** 10.3389/fonc.2025.1481952

**Published:** 2025-05-16

**Authors:** Yu-Ting Yin, Chao Gui

**Affiliations:** ^1^ Department of Gastrointestinal Surgery, Hubei Cancer Hospital, Tongji Medical College, Huazhong University of Science and Technology, Wuhan, Hubei, China; ^2^ Department of Head and Neck Surgery, Hubei Cancer Hospital, Tongji Medical College, Huazhong University of Science and Technology, Wuhan, Hubei, China

**Keywords:** giant thyroid tumor, thyroid metastasis, clear cell renal cell carcinoma, surgery, case report

## Abstract

Thyroid metastases from clear cell renal cell carcinoma are rare. Although they often present in combination with primary thyroid tumors, they are difficult to distinguish from primary thyroid tumors on imaging and are easily misdiagnosed preoperatively. In this article, we report a case of clear cell renal cell carcinoma that metastasized to the thyroid gland. A 69-year-old woman had been diagnosed with a thyroid mass 8 years earlier, which gradually enlarged into a giant thyroid tumor. Tumor compression of the trachea caused intermittent wheezing, and the tumor protruded into the thoracic cavity. The patient had a history of right nephrectomy performed 17 years prior due to right kidney necrosis. Following the preliminary diagnosis of a thyroid tumor, we performed a total thyroidectomy. Histopathological examination revealed metastatic clear cell renal cell carcinoma. The postoperative recovery was good; the patient did not receive further treatment or show signs of recurrence during the 1-year follow-up period. This case illustrates that thyroid metastasis of clear cell renal cell carcinoma can occur many years later and may be present in conjunction with primary thyroid tumors, making preoperative diagnosis difficult. Thorough surgical excision can provide good treatment results. This case report aims to promote the understanding and diagnosis of thyroid metastasis in patients with clear cell renal cell carcinoma.

## 1 Introduction

Metastatic disease is uncommon in the thyroid, accounting for 0.36–2.1% of all thyroid malignancies (1). Metastatic disease of the thyroid gland occurs in 1.4–3% of patients who undergo surgery for thyroid tumors. The most common primary sites of thyroid metastases tumors are the lungs, kidneys, head and neck, and breasts, with occasional reports of colorectal adenocarcinoma (2). Kidney cancer is the most common malignancy to metastasize to the thyroid gland, accounting for >50% of all clinically identifiable thyroid metastases (3). Renal cell carcinoma (RCC) accounts for 90% of all renal tumors and 3% of adult malignancies (4). RCC includes different subtypes; among them clear cell renal cell carcinoma (ccRCC) is the predominant pathological type, accounting for nearly 80% of all renal cancer cases (5).

Thyroid metastases are clinically insidious, lack specificity, and need to be differentiated from primary thyroid tumors. Local invasion of the thyroid tumor can lead to symptoms, such as dysphonia, dysphagia, dyspnea, hemoptysis, and hoarseness. When presenting as limited thyroid masses and in patients with an unknown history of primary malignancy, such lesions may pose a diagnostic challenge. Conventional diagnostic techniques cannot distinguish between primary and secondary thyroid tumors. Thyroid metastases can co-exist with primary thyroid disease, thereby complicating diagnosis. In 2012, a systematic review found that 44.2% of secondary thyroid malignancies occurred in abnormal glands with primary thyroid tumors or benign disease (6).

Although distant metastases are typically an indicator of poor prognosis, thyroid metastases may not necessarily be associated with such poor outcomes (7). The prognosis largely depends on the characteristics of the primary malignancy and the presence of systemic metastases at the time of diagnosis. Surgical removal of the thyroid gland is currently considered the best available treatment for metastatic kidney cancer to the thyroid gland (8).

In this article, we report a rare case of giant thyroid tumor compressing the trachea, which was considered a primary thyroid tumor prior to surgery. We performed total thyroidectomy, which resulted in the resolution of symptoms. Postoperative pathological analysis confirmed the diagnosis of metastatic ccRCC. There was no detection of tumor recurrence during the postoperative follow-up period.

## 2 Case presentation

A 69-year-old woman had been diagnosed with a neck mass 8 years earlier. However, she did not receive treatment or undergo further examination owing to the absence of other symptoms. The neck mass exhibited gradual growth, causing intermittent wheezing. Symptoms worsened over the last 3 months, with wheezing being more pronounced after activity. Consequently, she visited the hospital for treatment. This patient had undergone a right nephrectomy in a local hospital 17 years ago due to right kidney necrosis. Due to the limitations of medical technology at that time, there were no pathology results, and the patient did not undergo any other treatments or regular review. She denied previous use of tobacco, alcohol, or illegal substances. She has no family history of cancer or genetic disorders, and no other underlying conditions.

Physical examination revealed a palpable large mass (approximately 6 cm × 5 cm) in the right thyroid lobe and a palpable mass (approximately 3 × 2 cm) in the left thyroid lobe, which moved upward and downward with swallowing, was not tender, and was slightly hard.

Laboratory testing (i.e., renal function, liver function, blood, and urine) yielded results within normal limits. Thyroid function testing showed high thyroglobulin 264 ng/ml (normal value reference range: 3.5–77 ng/ml); nevertheless, the rest of the thyroid function results were normal. Considering the large size of the tumor, we advised the patient to undergo thyroid fine needle aspiration cytology (FNAC). However, she refused and requested to undergo surgery for the removal of the thyroid tumor tissue.

**Figure 1:**
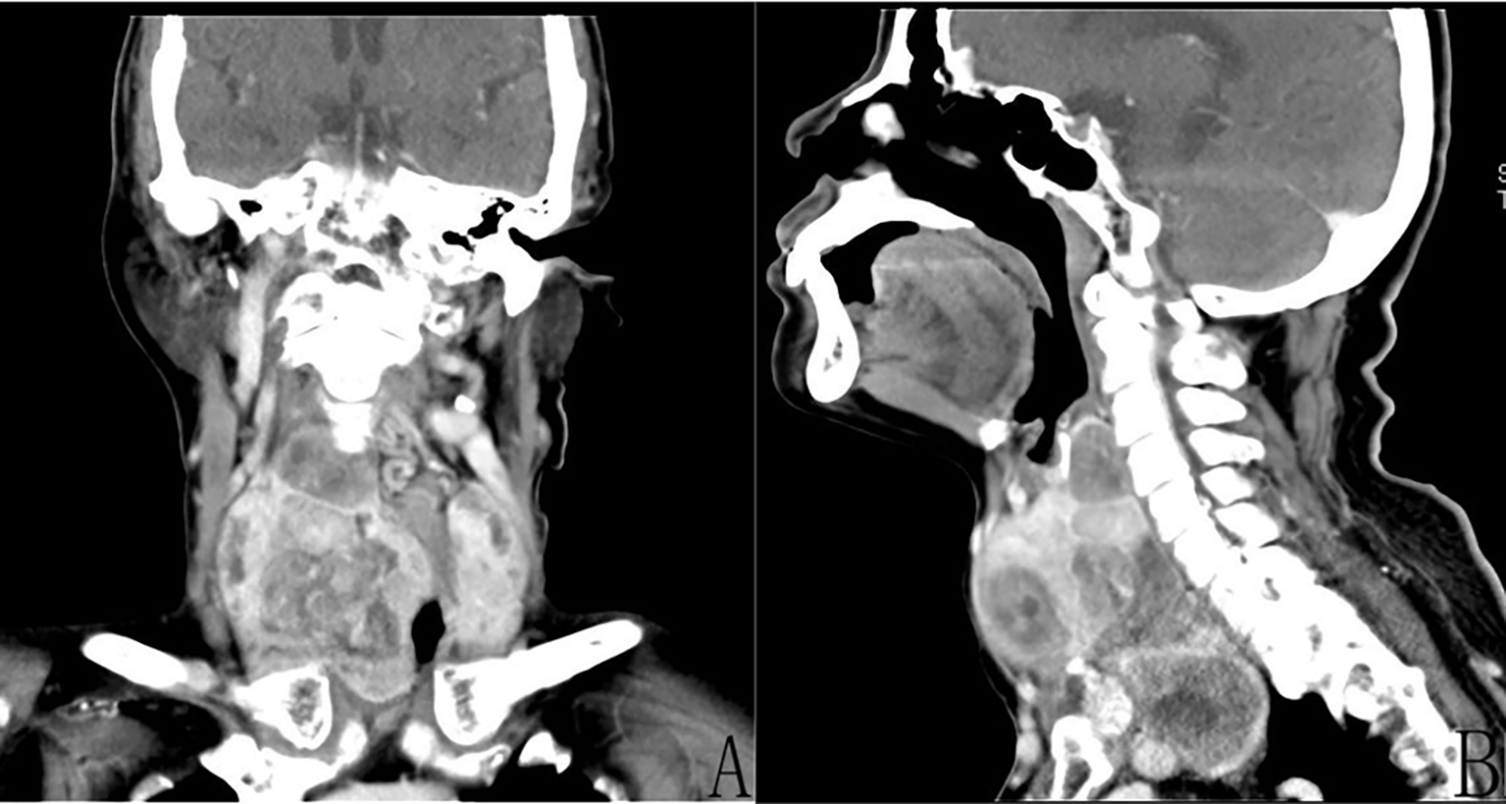
Enhanced computed tomography (CT) of the neck demonstrated the presence of multiple tumors of the thyroid gland bilaterally and inhomogeneous enhancement. **(A)** Coronal CT revealed a huge (approximately 8.4 cm × 8.3 cm) right thyroid tumor. **(B)** Sagittal CT revealed that the lower pole of the right tumor was located behind the sternum.

Computed tomography (CT) examination of the neck (**Figure 1**) showed significant increase in the size of the thyroid gland bilaterally (more pronounced on the right side: **Figure 1A**), with multiple low-density mass shadows accompanied by punctate calcification, markedly inhomogeneous enhancement, with a maximum cross-sectional size measuring approximately 8.4 cm × 8.3 cm, and with the lower pole of the mass growing into the posterior sternum (**Figure 1B**). Ultrasound examination of the abdomen showed normal liver, spleen, and pancreas, while stones (0.4 cm × 0.3 cm) were observed in the gallbladder. Moreover, the size and shape of the left kidney were normal; as mentioned above, the right kidney had been previously resected. Lung CT scan showed scattered mild chronic inflammation in the lungs with no sign of a tumor. Laryngoscopy showed normal vocal cord activity bilaterally. After ruling out contraindications to surgery, we instituted surgical treatment.

## 3 Gross findings of intraoperative tumors

**Figure 2:**
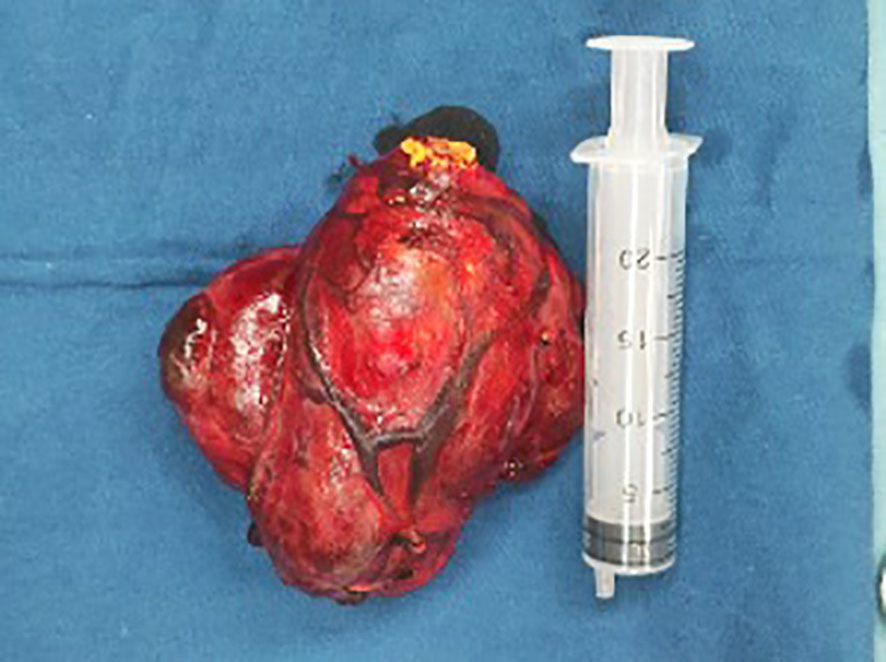
The thyroidectomy tissue envelope was intact, and golden nodules were observed.

Multiple thyroid masses were found on both sides. The right thyroid mass was huge, and the lower pole of the mass was located behind the sternum. The carotid sheath was pushed to the lateral side, showing that the mass had an intact envelope and was well-defined. The isthmus of the thyroid gland was severed, the thyroid envelope was opened, the upper pole of the thyroid gland was isolated and exposed, and the superior thyroid artery and accompanying vein were ligated. The thyroid gland was turned up from the outside in and the lateral vessels were coagulated. The recurrent laryngeal nerve was carefully located along the tracheoesophageal groove and protected under direct vision. The parathyroid glands were then identified and freed from the dorsal side of the thyroid gland. The thyroid was dissected and loosened strictly along the thyroid envelope to avoid injury to the carotid sheath. The retrosternal thyroid was gradually elevated after detachment and release along the thyroid envelope. The right thyroid and isthmus were excised (**Figure 2**), the tumor capsule was intact, with prominent blood vessels on its surface and yellow nodules observed at the apex. Rapid frozen pathological examination showed nodular goiter with multifocal clear cell lesions enriched in thin-walled blood sinusoids. Metastasis of renal carcinoma could not be excluded, and routine pathological and immunohistochemical examinations were performed to confirm the diagnosis. The left thyroid gland was isolated and exposed, the recurrent laryngeal nerve and parathyroid glands were found and protected, and the left thyroid gland was removed. The pathological results of the analysis of frozen sections showed nodular goiter. Metastasis of renal cancer could not be ruled out. The patient recovered well after the operation without complications (e.g., hoarseness, numbness of hands and feet).

## 4 Pathological findings

**Figure 3:**
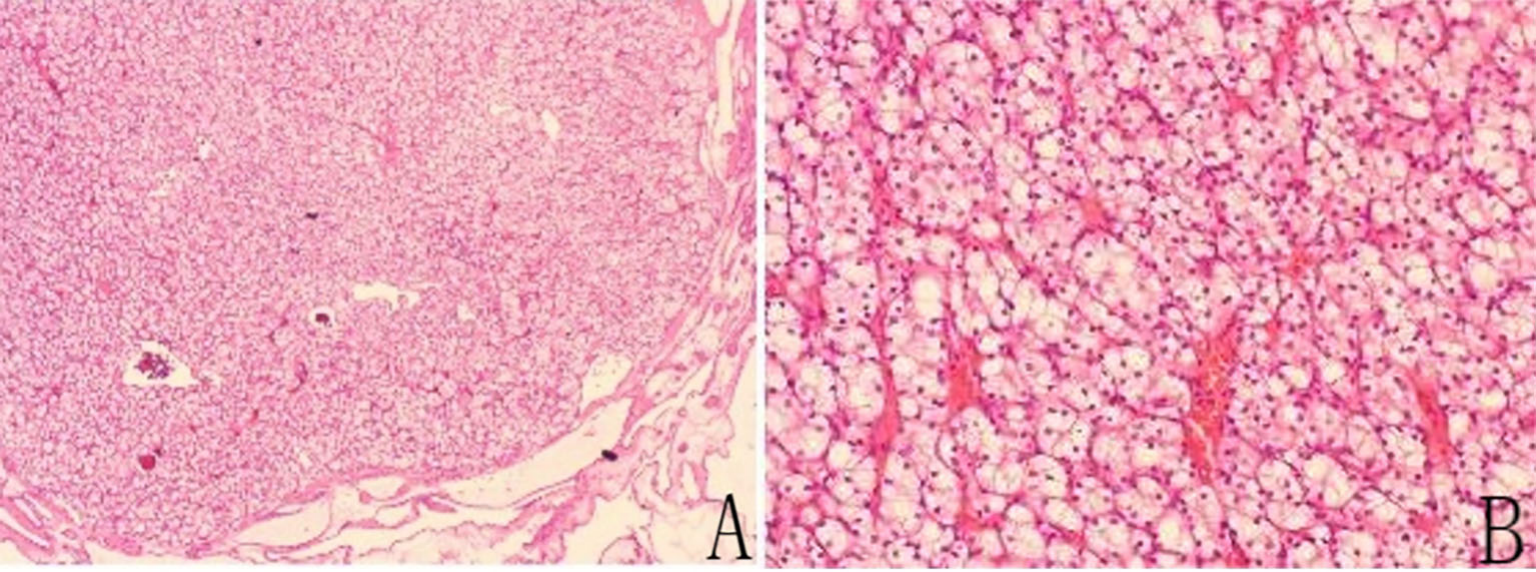
**(A)** Hematoxylin-eosin (HE) staining of a tumor specimen with thyroid tissue located externally below. Low magnification: HE × 40. **(B)** HE staining of the tumor specimens. High magnification: HE ×400.

Routine pathological findings (**Figure 3**) showed that both thyroid glands had metastatic ccRCC (World Health Organization/International Society of Urologic Pathologists grade 2) and nodular goiter. Immunohistochemical results revealed the following: paired box 8 (PAX8) (+); paired box 2 (PAX2) (+), vimentin (VIM) (+), CD10 (+), carbonic anhydrase IX (CAIX) (+), cytokeratin 7 (CK7) (−), thyroid transcription factor 1 (TTF1) (−), thyroglobulin (TG) (−), GATA binding protein 3 (GATA3) (−), synapsin (SYN) (−), chromogranin A (CgA) (−), and Ki67 (Clone: MIB E3 ubiquitin protein ligase 1 [MIB1]) (10%).

Postoperatively, the patient underwent a detailed systemic examination, including abdominal CT, whole-body bone scan, breast ultrasound, and cranial magnetic resonance imaging. These were performed in combination with preoperative lung CT, neck CT, and abdominal ultrasound, which did not indicate the presence of tumors in other sites. Long-term postoperative treatment with oral thyroxine tablets was administered to replace thyroid function. She did not receive further treatments (e.g., targeted therapy, radiotherapy, or chemotherapy) after surgery due to financial difficulties. At the 1-year postoperative follow-up, there were no signs of local recurrence or distant metastasis.

## 5 Discussion

Although the thyroid is a highly vascularized organ, it is a rare site of metastasis. This may be attributed to the rapid arterial blood flow, high oxygen saturation, and high iodine concentration, which inhibit the growth of malignant cells (9). Metastatic tumors account for a small proportion of thyroid cancers (≤2.1%) (1). Although the incidence is low, clinicians must consider the possibility of thyroid metastases in the routine diagnosis and management of thyroid nodules, particularly in patients with a history of cancer.

The most commonly reported primary tumor sites are the kidneys (25%), lung (22%), gastrointestinal tract (13%), breasts (13%), head and neck (5%), skin (4%), and other sites (10). The most common sites of metastasis for RCC include the lungs (10–57%), followed by the bone (16–27%), local sites (i.e., adrenal, retroperitoneal, renal fossa) (3–27%), liver (1–12%), and brain (2–4%) (11). According to the data, the thyroid gland is not a common site of metastasis for RCC. Approximately 90% of all distant metastatic renal carcinomas are considered metastases of ccRCC (12). Notably, ccRCC is rich in capillaries and blood sinuses; therefore, it is associated with a high risk of hemorrhage, necrosis, cystic degeneration, and calcification (13).

The present case underwent a right nephrectomy 17 years ago due to necrosis of the right kidney. Despite the lack of pathological results at that time, it is now assumed that the pathology was indicative of ccRCC. Golden nodules (**Figure 2**) were observed in the resected thyroid metastasis specimen, which may be related to the adequate blood supply.

Numerous patients with thyroid metastases exhibit the same signs and symptoms as those with primary thyroid disease. Thyroid metastases from RCC tend to be asymptomatic neck masses, typically appearing 2–7 years (or even 24 years) after surgery (14). The main route of metastasis is hematogenous. This patient had a metastatic thyroid tumor associated with a nodular goiter, presenting as a giant thyroid tumor. Metastases are common in patients with coexisting thyroid lesions, such as thyroiditis, goiter, and adenoma (15).

Metastatic RCC to the thyroid gland is frequently (42%) accompanied by adenoma or thyroiditis (16). Studies have found that diseased or abnormal thyroid glands are more prone to metastatic growth. This may be due to changes in metabolism, reduced levels of oxygen and iodine, and disruption of blood flow (17).

Common screening methods for thyroid metastases include ultrasound, FNAC, immunohistochemistry analysis, and positron emission tomography–CT. The common ultrasound features of thyroid metastases include nonspecific findings, such as inhomogeneous hypoechoicity, poorly defined borders, the absence of calcification, and increased vascularity. Thyroid metastases manifest ultrasound characteristics analogous to those observed in primary thyroid tumor (18). FNAC is commonly used in the preoperative evaluation of thyroid nodules to differentiate between benign and malignant thyroid nodules. Patients with a history of cancer, especially renal cancer, may obtain histopathology by fine needle aspiration biopsy or surgical resection to avoid missed diagnosis of thyroid metastases. Nonetheless, its diagnostic accuracy for thyroid metastases is limited, particularly in patients with a combined primary thyroid tumor. In a study using FNAC to diagnose metastatic RCC, the rate of false-negative results was 28.7% (6). FNAC combined with immunohistochemistry analysis is highly accurate for diagnosing thyroid metastases and can also differentiate the primary site of the tumor (19). This patient was positive for renal markers (i.e., PAX8, PAX2, CD10, CK7) and negative for thyroid markers (i.e., TTF1, TG). The morphological and immunohistochemical features of the tumor, as well as her history of nephrectomy, were indicative of metastatic ccRCC. The recommendation for FNAC of new thyroid nodules in patients with cancer is to puncture a sample sufficiently and perform immunohistochemistry to identify the primary cancer and metastasis.

This was a giant thyroid tumor, and its lower pole was located behind the sternum. Thus, ultrasound could not detect the full extent of the tumor; consequently, we opted for CT of the neck. Enhanced CT and three-dimensional reconstruction of the neck can provide a relatively comprehensive image of the thyroid tumor and valuable details of the adjacent anatomical structures. This allowed us to select the surgical approach. The CT image showed a giant tumor with clear boundaries and an intact envelope. Thus, it was possible to completely remove the tumor tissue from the neck through an incision.

Secondary thyroid tumors are insensitive to treatment with radioactive iodine (11). RCC has limited sensitivity to chemotherapy, and the cornerstone of RCC treatment currently lies in targeted therapies and immunotherapy (20). Sunitinib may be effective in the treatment of such metastases (21). The availability of new effective drugs increases the options for the treatment of patients with metastatic RCC. Thyroidectomy is considered a more effective treatment modality than radiotherapy and chemotherapy (22).

Metastatic renal cancer is linked to a poor prognosis, with a median overall survival of 2 years and a 5-year survival rate of 12%. Removal of isolated metastases markedly improves the prognosis; it was previously shown that removal of isolated thyroid metastases increased the 5-year survival rate to 50% (3). Russell et al. reported that patients who undergo thyroidectomy, particularly those with metastatic RCC, have longer survival; this may be related to the fact that RCC is less aggressive (23). Koo et al. reported a case of thyroid metastasis 5 years after surgery for RCC. Thyroid metastases were treated with immunotherapy, and there was no sign of recurrence at the 3-month postoperative follow-up (24). Gawlik et al. reported a case of ccRCC that metastasized to the thyroid gland. The patient did not receive adjuvant therapy after surgery and was regularly followed up with no recurrence of the tumor (25). However, Lee et al. showed that thyroid metastases from RCC had the worst prognosis because most patients had multiple metastases involving the thyroid gland rather than isolated metastases (26).

When a tumor that metastasized to the thyroid is sufficiently large to cause compression symptoms, thyroidectomy may be considered for symptom relief. When the thyroid is the only site of metastasis, the primary tumor is of low malignancy, and the patient is associated with long survival. In such cases, surgery may achieve a long-term cure. This patient was suffering from intermittent shortness of breath due to a huge thyroid tumor compressing the trachea. It had been 17 years since the resection of the primary renal cancer. After a thorough examination of the whole body to confirm that only thyroid metastasis was present, she was cured by complete excision of the metastatic thyroid tumor. Patients with RCC have a longer postoperative survival rate and are more likely to undergo further research and treatment. This patient did not receive adjuvant therapy after surgery for financial reasons. Furthermore, although no tumor recurrence was detected at the 1-year follow-up, close monitoring and regular review are still required.

This patient presented with bilateral thyroid nodules (the right nodule was giant); we performed a total bilateral thyroidectomy, and the postoperative follow-up was good. Regional lymphatic metastases should be thoroughly evaluated preoperatively. Prophylactic cervical lymphadenectomy is generally not recommended because regional lymph node involvement is uncommon (11). In this patient, preoperative examination did not reveal any obvious abnormalities in cervical lymph nodes. Thus, we did not perform a cervical lymph node dissection in conjunction with the results obtained from the intraoperative analysis of frozen sections.

Thyroid metastases can occur long after nephrectomy and portend a better prognosis (3). The prognosis largely depends on the characteristics of the primary malignancy and the presence of systemic metastases. This patient presented with oligometastases to the thyroid gland 17 years after undergoing right nephrectomy, and no further treatment was administered after these two surgeries. The patient recovered well after surgery, and there was no tumor recurrence during the follow-up.

## 6 Conclusion

Thyroid metastases from ccRCC are exceedingly rare, with a low incidence of growth into giant thyroid tumors. This case demonstrates that a metastatic thyroid tumor from renal cancer may coexist with primary thyroid tumors, and giant thyroid tumors may comprise multiple tumor components, which poses a challenge for preoperative diagnosis and is difficult to differentiate on imaging. In such cases, histopathological sections and immunohistochemical analysis are often required to confirm the diagnosis. It is imperative to consider the possibility of thyroid metastasis in patients with a history of cancer and a thyroid tumor. ccRCC can induce oligometastases of the thyroid many years after the primary tumor resection, which can be effectively treated with a total thyroidectomy.

## Data Availability

The original contributions presented in the study are included in the article/supplementary material. Further inquiries can be directed to the corresponding author.
